# The impact of same-day antiretroviral therapy initiation on retention in care and clinical outcomes at four eThekwini clinics, KwaZulu-Natal, South Africa

**DOI:** 10.1186/s12913-023-09801-0

**Published:** 2023-08-08

**Authors:** Sabina M. Govere, Chester Kalinda, Moses J. Chimbari

**Affiliations:** 1 School of Nursing and Public Health, Discipline of Public Health Medicine, Howard College Campus, University of KwaZulu-Natal, Durban, South Africa; 2 Bill and Joyce Cummings Institute of Global Health and Institute of Global Health Equity Research (IGHER), University of Global Health Equity Kigali Heights, Kigali, Rwanda; 3 Department of Public Health, Great Zimbabwe University, P.O Box 1235, Masvingo, Zimbabwe

**Keywords:** Same day ART initiation, Retention in care, ART adherence, Clinical outcomes

## Abstract

**Background:**

Same-day initiation (SDI) of antiretroviral therapy (ART) increases ART uptake, however retention in care after ART initiation remains a challenge. Public health behaviours, such as retention in HIV care and adherence to antiretroviral therapy (ART) pose major challenges to reducing new Human Immunodeficiency Virus** (**HIV) transmission and improving health outcomes among HIV patients.

**Methods:**

We evaluated 6-month retention in care, and clinical outcomes of an ART cohort comprising of SDI and delayed ART initiators. We conducted a 6 months’ observational prospective cohort study of 403 patients who had been initiated on ART. A structured questionnaire was used to abstract data from patient record review which comprised the medical charts, laboratory databases, and Three Interlinked Electronic Registers.Net (TIER.Net). Treatment adherence was ascertained by patient visit constancy for the clinic scheduled visit dates. Retention in care was determined by status at 6 months after ART initiation.

**Results:**

Among the 403 participants enrolled in the study and followed up, 286 (70.97%) and 267 (66.25%) complied with scheduled clinics visits at 3 months and 6 months, respectively. One hundred and thirteen (28.04%) had been loss to follow-up. 17/403 (4.22%) had died and had been out of care after 6 months. 6 (1.49%) had been transferred to other health facilities and 113 (28.04%) had been loss to follow-up. Among those that had been lost to follow-up, 30 (33.63%) deferred SDI while 75 (66.37%) initiated ART under SDI. One hundred and eighty-nine (70.79%) participants who had remained in care were SDI patients while 78 (29.21%) were SDI deferred patients. In the bivariate analysis; gender (OR: 1.672; 95% CI: 1.002–2.791), number of sexual partners (OR: 2.092; 95% CI: 1.07–4.061), age (OR: 0.941; 95% CI: 0.734–2.791), ART start date (OR: 0.078; 95% CI: 0.042–0.141), partner HIV status (OR: 0.621; 95% CI: 0.387–0.995) and the number of hospitalizations after HIV diagnosis (OR: 0.173; 95% CI: 0.092–0.326). were significantly associated with viral load detection. Furthermore, SDI patients who defaulted treatment were 2.4 (95% CI: 1.165–4.928) times more likely to have increased viral load than those who had been returned in care.

**Conclusion:**

Viral suppression under SDI proved higher but with poor retention in care. However, the results also emphasise a vital need, to not only streamline processes to increase immediate ART uptake further, but also to ensure retention in care.

## Introduction

Availability of Antiretroviral therapy (ART) to individuals diagnosed with Human Immunodeficiency Virus (HIV) has led to improved disease prognosis, healthier quality of life and reduction of HIV transmission [1]. However, treatment adherence and retention in care among people living with HIV remains fundamental in attaining these outcomes [2, 3]. In 2015, the World Health Organization (WHO) recommended ‘same-day ART initiation’ (SDI) of antiretroviral therapy (ART) under the Universal Test and Treat (UTT) policy for people living with HIV (PLHIV) [4, 5]. Many countries in sub-Saharan Africa (SSA) including South Africa, have introduced the SDI into their national HIV guidelines [6–8]. HIV-positive individuals who adhere to treatment and are retained in care can suppress the HIV viral level in their serum to undetectable levels, thus eliminating the risk of transmitting HIV to others [9, 10]. However, WHO noted that the anticipated achievements of such an approach could only be accomplished if improvements were made in retaining patients in care [11].

The Joint United Nations Programme on HIV/AIDS (UNAIDS) and World Health Organization (WHO) introduced the 95-95-95 initiative to further decrease new infections globally [12]. The initiative maximizes the effect of ART coverage by emphasizing that 95% of HIV-positive people should know their status, 95% of those eligible for ART should be initiated on ART, and 95% of those on ART should achieve and maintain viral suppression [12, 13]. However, sub-Saharan Africa (SSA) region may miss the UNAIDS) 95-95-95 target of reducing HIV epidemic by 2030 [14]. By the end of 2019, SSA had reported that 77% of PLHIV knew their HIV status, 72% of those diagnosed were initiated on ART and only 65% of those on ART achieved sustaining viral load suppression [15]. Retention in HIV care intensely affects HIV disease outcomes at individual and population levels [16, 17]. Currently, retention in HIV care programmes has varied widely worldwide [18]. In 2022 statistics, the global retention in HIV treatment management was 90% at 6 months and 84% at 12 months [19].

The 2022 South Africa report on 95-95-95 targets indicates relative success with HIV testing, ART initiation and viral suppression, achieving 94% and 76% success rates and 89% respectively [19]. It is well documented that young people aged less than 30 years and men, are being lost to follow-up along the entire HIV care cascade [20]. Furthermore, most individuals are lost in the HIV care cascade between HIV diagnosis to the start of treatment [21]. Effective care of people living with HIV/AIDS (PLWH) requires that patients are provided with satisfactory care, adhere to their treatment regimen, and are retained in care [6, 22].

In the field of HIV medicine, patients who receive regular medical care and attend scheduled clinic visits are considered retained in care [23]. Retention in care is not only important for the individual health of people living with HIV, but also for public health Accordingly, retention in care is a critical pillar of public health strategy to eliminate HIV transmission. Our study assessed the association between retention in care, clinical outcomes, and predictors of attrition particularly mortality and loss to follow up by comparing individuals who accepted same day ART initiation and those who deferred same day ART initiation.

## Materials and methods

### Study design and population

This prospective cross-sectional study was conducted at 4 clinics in eThekwini municipality KwaZulu-Natal (KZN), South Africa between June 2020 to December 2020. The study sites were Ithembalabantu, Pinetown, D, and Qadi clinic. KZN has 1.9 million people living with HIV of which only 1.1 million are on ART [12]. Of the estimated 650,000 people living with HIV in eThekwini, there are approximately 383,869 people in the ARV program [24]. The eThekwini district is densely populated (3,702,231) comprising of urban, semi-urban, and rural areas [24]. We selected study clinics from the three settings; (i) 2 facilities (Ithembalabantu and D clinic) in a densely populated Umlazi township also known to have high HIV prevalence of 35%, [25] (ii) Pinetown clinic in Pinetown, a semi suburb town (these are places that offer a balance between township and suburb tranquillity) surrounded by townships and informal settlements who all seek services at the facility and (iii) Qadi clinic in rural Umzinyathi district municipality north of eThekwini municipality. UMzinyathi district has high levels of poverty, unemployment, and HIV/AIDS [25].

We enrolled 403 participants from June to November 2020 meeting the eligibility criteria for the national guidelines on ART initiation. The guidelines state that all HIV-positive diagnosed individuals should rapidly initiate on ART regardless of CD4 count, receive treatment adherence counselling, those not ready to start ART and Tuberculosis asymptomatic awaiting for TB results initiate later as per program guidelines. The study participants were followed up for a period of six months after ART initiation from December 2020 to May 2021.

### Data collection

A structured questionnaire was used to extract information on patient adherence to treatment, and retention in care and clinical outcomes using patient chart reviews. Data were collected electronically using the Kobo Collect application on Android mobile devices [26]. Data was collected from HIV patient medical charts, National Health Laboratory Services (NHLS) database for specimen results and Three Interlinked Electronic Registers.Net (TIER.Net). Routinely collected HIV patient level data that was entered into the electronic medical record system, called Three Interlinked Electronic Registers.Net (TIER.Net), an electronic ART database developed by the University of Cape Town’s Centre for Infectious Disease Epidemiology and Research. We extracted individual demographic characteristics, clinic visit dates, recorded comorbidities, specimen results and clinical outcomes particularly viral load results at 6 months visit after ART initiation, however knowledge of UTT and ART was obtained from administered questionnaire during interviews.

### Data analysis

Adherence to treatment was ascertained by consistently attending scheduled patient visit dates for collection of medication both in the clinic and external medication pick up points. Retention in care was determined by clinic visits status at 6 months after ART initiation. Lost to follow-up was defined as no documented death or transfer and no clinical visit or pharmacy pick-up during the last 90 days according to the South Africa guidelines. Data collected was analysed using Stata version 17.0 (College Station, TX, USA). Descriptive and bivariate analysis were used to summarize socio-demographic characteristics of the participants as well as evaluating their association with the study outcome which was viral load detection and retention in care. Bivariate associations between each independent variable and viral load detection were determined using unadjusted logistic regression model, and variables associated with viral load detection at the level of p ≤ 0.2 were included in the multivariable logistic model. Viral load detection was associated to a number of factors with some of the factors also correlated with CD4 count. Therefore, our analysis considered all these factors in the model to minimize confounding effects. To achieve this, we estimated a step-wise model building for our outcome on the initial model by adding more controls at each stage (First level model: unadjusted (socio-demographic characteristics of participants); Second level model: added health outcomes and haematology laboratory results weight; Final-model: adjusted for date of start of ART. We also tested for interaction and for clustering by hospital site in order to fit it as a random effect. However, when site was included as our random effect to allow for robust estimate of viral load detection within and between the study clinics there, was no evidence of interaction. Goodness of fit and model adequacy were tested using the fit is the Hosmer–Lemeshow test [27]. The primary outcomes were retention in care and viral suppression at 6 months after ART initiation. Retention in care was defined as consistently attending all (1, 3 and 6 months) scheduled clinic visits for treatment collection and clinical management. Viral suppression was defined as ≤50 copies/ml considered as viral suppressed within 6 (months of ART initiation, a time period selected to capture the 6 months routine monitoring visit according to ART national guidelines. Secondary outcomes evaluated in the study included the prevalence of comorbidities, hospitalisations, demographic predictors and level of CD4 results.

## Results

### Participants characteristics at the time of ART initiation

All 403 study participants were followed up using routine health data collected in TIER.NET and medical charts for 6 months from the date of HIV diagnosis. Two hundred and eighty-six (70.97%) and 267 (66.25%) visited scheduled clinics at 3 months and 6 months, respectively. Among those that had missed their scheduled visit dates after six months, 17 (4.22%) had died, 6 (1.49%) had been transferred to other health facilities and 113 (28.04%) had been loss to follow-up. Among those that had been lost to follow-up, 30 (33.63%) deferred SDI of ART while 75 (66.37%) initiated ART under SDI. Participants who had remained in care, 189 (70.79%) were SDI patients while 78 (29.21%) were SDI deferred patients (Table 1). Among the 189 SDI patients, 178 (94.18%) had their viral load undetected while 11 (5.82%) had a detectable viral load. In addition, the SDI deferred patients, 29 (37.18%) had their viral load suppressed while 49 (62.82%) had a detectable viral load. However, there was no evidence (*p*=0.343) of association between viral load suppression and time of ART initiation when controlled for remaining in care or not.

**Table 1:** Socio-demographic characteristics of patients based on their viral load detection

**Viral load**					
**Undetectable**	**Detectable**				***p-value***
Variables	(N)	%	Freq (N)	%	
**Age**					
18-28	65	69.15	29	30.85	0.820
29-39	92	71.88	36	28.12	
40-50	42	76.36	13	23.64	
51-62	14	70	6	30	
**Gender**					
Female	138	75.82	44	24.18	**0.048**
Male	75	65.22	40	34.78	
**Marital status**					
Cohabiting	39	72.22	15	27.78	0.307
Divorced	5	55.56	4	44.44	
Married	54	73.97	19	26.03	
Single	98	69.01	44	30.99	
Widowed	17	89.47	2	10.53	
**Education Level**					
Primary	54	70.13	23	29.87	0.912
Matric	98	69.01	35	27.13	
Diploma	65	71.43	26	28.57	
**Employment status**					
Employed	75	74.26	26	25.74	0.826
Self Employed	18	72	7	28	
Student	19	65.52	10	34.48	
Unemployed	101	71.13	41	28.87	
Biological children					
No	40	64.52	22	35.48	0.157
Yes	173	73.62	62	26.38	
**Knowledge of Universal Test and Treat (UTT)**					
No	151	51.01	90	84.11	**0.001**
Slightly	47	15.88	11	10.28	
Yes	98	33.11	6	5.61	
**Number of sexual partners**					
One	59	81.94	13	18.06	**0.027**
More than 2	154	68.44	71	31.56	
**HIV status of sexual partner**					
Negative	6	75	2	25	**0.069**
Unknown	108	66.26	55	33.74	
Positive	99	78.57	27	21.43	
**Tested positive for HIV before**					
No	176	70.12	75	29.88	0.153
Yes	37	80.43	9	19.57	
**Time between HIV test and initiation**					
Later	30	34.48	57	65.52	**0.000**
Same Day	183	87.14	27	12.86	
**Clinic scheduled visit at 1 month**					
Yes	211	71.53	84	28.47	0.373
No	0	100	0	0	
**Clinic scheduled visit at 3 month**					
Yes	208	76.19	65	23.81	**0.000**
No	5	20.83	19	79.17	
**Clinic scheduled visit at 6 month**					
Yes	207	77.53	60	22.47	**0.000**
No	20	20	24	80	
**Patient still in care**					
Yes	207	77.53	60	22.47	**0.001**
No	5	38.46	8	61.54	
**Infections during the study period (6 months)**					
Yes	53	39.26	82	60.74	**0.000**
No	160	98.77	2	1.23	
**Number of hospitalisations**					
None	4	23.53	13	76.47	**0.000**
One	3	15	17	85	
Two	206	79.23	54	20.77	

Although there was no association between viral load and age (*p*=0.820), most respondents aged between 29- 39 years 71.8% (*n*=92) years had an undetectable viral load. We also observed a significant association with gender with 75.8% (*n*=138) of the females had suppressed viral load. The majority 69.1% (*n*=98) of the respondents were single and of these, 69.0% (*n*=98) had an undetectable viral load. Among the (*n*=142) unemployed patients, we observed that 71.1% (*n*=101) had an undetectable viral load. We observed that there was a correlation between viral load and the number of sexual partners, those with one sexual partner were more likely to have a suppressed viral load compared to those with more than two sexual partners (*p*=0.027). There was a significant association between having no knowledge of UTT and a detectable viral load (*p*=0.001). Participants who initiated ART on the same day of HIV diagnosis were more likely to have an undetectable viral load compared to those who delayed ART initiation (p-0.000). Factors such as HIV status of partner, scheduled clinic visits at 3 months after initiation, remaining in care (6 months after initiations), number of hospitalisations after testing were all associated with viral load suppression (*p*<0.05) (Table 1).

### SDI initiation and its association with CD4 count and co-infections

Forty-five point seven percent (45.7%; *n*=184) of the respondent had contracted another disease during the six months’ follow-up period. Of the 403 56.5% (*n*=104) were SDI deferred patients while 43.5 (*n*=80) (*p*=0.001) were SDI. The most common coinfection contracted by both the SDI (n=44) and SDI deferred patients (*n*=49) was STI shingles. On the other hand, 42.3% (*n*=44) of the SDI deferred patients had TB while only 1.25% (*n*=1) of the SDI patients had TB. We also observed that 9.78% (*n*=18) of the SDI patients had contracted hypertension while non-of the SDI deferred patients had contracted the same. Furthermore, 12.5% (*n*=10) and 7.5% (*n*=5) of the SDI patients had contracted Covid-19 and Cryptococcus Meningitis respectively while 5.8% (*n*=6) and 2.9% (*n*=3) of the SDI deferred patients had contracted the same diseases.

In addition, we observed that among the SDI patients, 65.2 (*n*=182) had a CD4 count of >350 copies/ml and 34.8% (97%) had a CD4 count of <350 copies/ml. On the other hand, among the SDI deferred patients, 37.9% (*n*=47) and 62.1% (*n*=77) had >350 copies/ml and <350 copies/ml CD4 count respectively. We also observed that of the 78 SDI deferred patients who had remained in care, 33.3% (*n*=26) had a CD4 count of >350 copies/ml while 66.7% (*n*=52) had a CD4 count <350 copies/ml. On the other hand, of the 189 SDI patient, 82% (*n*=155) had a CD4 count above 350 copies/ml while 18% (*n*=34) had a CD4 count below 350 copies/ml.

### Factors associated with viral load suppression 6 months after initiation

Among the variables observed to be associated with the viral load detection in the bivariate analysis; male gender (OR: 1.672; 95% CI: 1.002–2.791), more than two sexual partners (OR: 2.092; 95% CI: 1.07–4.061), 18-28 years of age (OR: 0.941; 95% CI: 0.734–2.791), ART start date (OR: 0.078; 95% CI: 0.042–0.141) and partner HIV status (OR: 0.621; 95% CI: 0.387–0.995) were significantly associated with viral load detection. Missing clinic scheduled visits at 3 months (OR: 1.16; 95% CI: 1.368-4.384) and not remaining in care (OR: 3.52; 95% CI: 1.743–5.498) (Table 2).

**Table 2:** Factors associated with viral load suppression

**Determinant**	**OR (Unadjusted)**	**95% CI**	***P*-value**
**Age**			
18-28	Reference		
29-39	0.877	0.489–1.571	**0.629**
40-50	0.693	0.324–1.484	
51-62	0.96	0.335–0.691	
**Gender**			
Female	Reference		**0.049**
Male	1.67	0.546–1.37	
**Marital status**			
Married	Reference		
Single	0.96	0.78–1.18	0.683
**Employment status**			
Employed	Reference		0.566
Unemployed	1.06	0.88–1.27	
**Biological children**			
No	Reference		0.159
Yes	0.65	0.36– 1.18	
**Number of sexual partners**			
One	Reference		**0.029**
More than 2	2.09	1.08–4.06	
**HIV status of sexual partner**			
Negative	Reference		**0.048**
Positive	0.62	0.39–0.99	
**Time between HIV test and initiation**			
Later	Reference		**0.000**
Same Day	0.08	0.04–0.14	
**Clinic scheduled visit at 3 months**			
Yes	Reference		**0.000**
No	1.16	1.03–4.85	
**Patient still in care**			
Yes	Reference		**0.023**
No	3.52	1.743–5.498	
**Infections during the study period (6 months)**			
Yes	Reference		**0.000**
No	1.277	29.42–520.68	
**Hospitalizations**			
Yes	Reference		**0.000**
No	0.17	0.09–0.33	

Our results showed that among those who did not remain in care (aOR: 2.44; 95% CI: 1.614–3.872) they reported having co-morbidities during the period of the study (aOR: 1.212; 95% CI: 1.031–1.425) which was a predictors of viral load detection. We also observed that the odds of viral load surge were 2.313 (95% CI: 1.591–4.987) higher among those not remaining in care than those who remained in care. In addition, patients with chronic conditions such as hypertension were 0.082 (95% CI: 0.008–0.257) times more likely to have reduced viral load than those with Covid-19 ( Table 3).

**Table 3:** Predictors of viral load suppression

**Determinant**	**OR (Unadjusted)**	**95% CI**	***p*-value**	**OR (adjusted)**	**95% CI**	***p*-value**
**Remain in care**						
Yes	Reference					
No	**3.52**	**1.743–5.498**	**0.023**	**2.313**	**1.591-4.987**	**0.001**
**Infections during the study period (6 months)**						
Covid-19	Reference					
**Hypertension**	**0.031**	**0.004-0.257**	**0.001**	**0.071**	**0.006-0.732**	**0.026**
STI shingles	0.905	0.724-1.074	0.831			
Tuberculosis	0.374	0.100-1.403	0.145			

## Discussion

In a cohort of HIV diagnosed individuals who chose to start or defer ART initiation under the UTT policy, we observed a gradual reduction in the number of patients who went for treatment collection at three months and six months after ART initiation. Earlier studies conducted such as the the Rapid Initiation of Treatment (RapIT) trial and the Simplified Algorithm for Treatment Eligibility (SLATE) study in South Africa and Kenya, demonstrated that SDI improved viral suppression rates but showed limited evidence for improved retention in care [28].

The increase in loss of patients to follow up among participants who initiated ART on the same day of HIV diagnosis we observed corroborates with results from the SLATE trial conducted in South Africa and Kenya that in that same-day treatment initiation increased rapid ART uptake but not necessarily retention in care especially in the early months after initiation [29]. Trends in South Africa indicate that individuals increased risk of being lost to follow-up at six months’ months as a result of accelerated ART initiation. Treatment readiness , pill burden and in some cases disclosure may contribute to increased loss to follow up [30].

Intensified patient education focussing on the benefits of ART initiation and consistent treatment is crucial to increase patient retention [31, 32]. Furthermore, additional treatment support such as weekly treatment literacy classes and continuous adherence counselling sessions are most relevant in the first six months of care to reduces cases of patient attrition which our data and other studies indicated that it is substantial in the first six months of care [33, 34]. In a study conducted by Pascoe, Fox [31], Fast‐Track Initiation Counselling (FTIC) had some short‐term but no long‐term benefits suggesting that for FTIC initiation had treatment adherence benefits, particularly in the test‐and‐treat era with higher chances of initiating individuals who are not psychologically ready for treatment. To achieve this, FTIC support post‐initiation may need to be strengthened and paired with other effective interventions designed to support patients with adherence and retention [31]. Consistent with previous studies, the time restrictions to initiate ART in line with SDI policy probably resulted in the overall decline in the quality of extensive post HIV counselling before ART initiation.

Our findings indicate that substantial effort is required to encourage treatment adherence and further sustain retention of PLHIV in care, especially during the first six months of ART initiation. Several, socio-demographic characteristics such as sex, gender, marital status, and employment status must be considered when developing policy and HIV care interventions since these pose technical hitches in effective policy implementation and sustainability on public health benefits.

We found that individuals who remained in care were more likely to have a suppressed viral load at 6 months. Unsuppressed viral load at 6 months was associated with sub-optimal ART adherence, and this is corroborated by findings in studies conducted in Nigeria and Malawi amongst individuals attending a public ART programme [2, 35]. According to these authors, patients who were tracked back into care after missing their scheduled visits had detectable viral loads and presented with comorbidities with some patients requiring hospitalisations [2, 35]. Their findings corroborated with our results which showed high reported cases of comorbidities, hospitalisations among patients with unsuppressed viral load with hypertension and tuberculosis being the common infections due to declining immune functioning.

We identified that SDI individuals were more likely to be virally suppressed compared to those that deferred ART at 6 months after ART initiation. We also found that individuals who deferred same day ART initiation had greater odds of getting opportunistic infections with the common infection being TB. This might have been caused by low CD4 count results in the deferred SDI group. These findings were consistent with a study conducted in Johannesburg South Africa which showed that individuals who delayed to initiate on ART later on presented with advanced HIV disease, co-infections and low CD4 counts which were complicated to manage [11] .

### Strengths and limitations of this study

Some of the strengths of our study include; i) being done in an urban and peri‐urban communities of KwaZulu-Natal, a province with large numbers of patients on treatment thus providing a reasonable basis for generalizability for many people living with HIV in South Africa and sub-Saharan Africa, ii) being able to analyse at 6 months after ART initiation which is crucial in clinical assessments to observe if patients are responding well to treatment. However, some of the weaknesses of our study include that the population comprised of adults only hence the results on retention in care, viral suppression and clinical outcomes may not apply to infants and children and the Tier.Net is individualised per facility implying that some patients might have self-transferred themselves to other clinics or migrated and not actually lost to follow up. This is because patients exiting care from the ART initiating clinic may re-enter care elsewhere (i.e., silent transfers) and appear as lost to follow-up when they are still in care.

## Conclusion

We found evidence of a significant relationship between SDI and viral suppression but poor retention in care during the first 6 months of ART initiation. Individuals who initiated ART on the same day of HIV diagnosis and remained in care showed clinically meaningful outcomes. The CD4 count results, suppressed viral load, and reduced incidence of co-infections supported “proof of principle” for same day ART initiation algorithm. This study also provides much needed evidence on the relationship between adherence and viral suppression in this setting and supports the 3^rd^ 95% of the UNAIDS 95-95-95 target. However, the results also emphasise a vital need, to not only streamline processes to increase immediate ART uptake, but also ensure retention in care. There is need to review the same day ART initiation policy considering the amount of time some individual require to process and accept a positive HIV diagnosis before committing to lifelong treatment. Given the high HIV prevalence in eThekwini municipality and South Africa in general, adequate staff provision especially trained professional healthcare workers need to be addressed to reduce time taken initiating ART and effective continuous treatment management including adherence counselling services.

## Data Availability

The datasets used and/or analysed during the current study are available from the corresponding author on reasonable request.

## Abbreviations

SDI: Same-day initiation
ART: Antiretroviral therapy
HIV: Human Immunodeficiency Virus
TIER.Net: Three Interlinked Electronic Registers.Net
WHO: World Health Organization
UTT: Universal Test and Treat
PLHIV: People Living with HIV
SSA: Sub-Saharan Africa
